# Risk factors associated with non-alcoholic fatty liver disease in subjects from primary care units. A case-control study

**DOI:** 10.1186/1471-230X-8-44

**Published:** 2008-10-03

**Authors:** Llorenç Caballería, Ma Antonia Auladell, Pere Torán, Guillem Pera, Dolores Miranda, Alba Alumà, José Dario Casas, Laura Muñoz, Carmen Sanchez, Albert Tibau, Marti Birules, Santiago Canut, Jesús Bernad, Josep Aubà, Miren Maite Aizpurua, Enriqueta Alcaraz

**Affiliations:** 1 Primary Healthcare Centre Premià de Mar, Catalan Health Institute, IDIAP Jordi Gol, La Plaça 93, 08330 Premià de Mar, Spain; 2 Primary Healthcare Research Support Unit Barcelonés Nord i Maresme. IDIAP Jordi Gol, Camí del Mig 36, 08303 Mataró, Spain; 3 Radiology Department, Primary Healthcare El Maresme, Catalan Health Institute, Camí del Mig 36, 08303 Mataró, Spain; 4 Laboratory Department, Primary Healthcare Centre Dr. Robert, Catalan Health Institute, C/Pl. de la Medicina s/n, 08911 Badalona, Spain; 5 Radiology Department, Primary Healthcare Badalona, Catalan Health Institute, C/Pl. de la Medicina s/n, 08911 Badalona, Spain; 6 Radiology Department, Primary Healthcare Santa Coloma de Gramanet, Catalan Health Institute, C/Major, 49-53, 08921 Santa Coloma de Gramanet, Spain; 7 Primary Healthcare Centre Premià de Mar, Catalan Health Institute, C/La Plaça 93, 08330 Premià de Mar, Spain; 8 Primary Healthcare Centre Poble Nou, Catalan Health Institute, C/Lope de Vega 132, 08005 Barcelona, Spain; 9 Primary Healthcare Centre Vilassar de Dalt, Catalan Health Institute, C/Plaça de la Vila 8, 08339 Vilassar de Dalt, Spain; 10 Primary Healthcare Centre Vilassar de Mar, Catalan Health Institute, C/Santa Maria 59-79, 08340 Vilassar de Mar, Spain; 11 Primary Healthcare Barcelonés Nord i Maresme, Catalan Health Institute, Sardana s/n, 08915 Badalona, Spain; 12 Primary Healthcare Centre Gatassa, Catalan Health Institute, Camí del Mig 36 (4a planta), 08303 Mataró, Spain

## Abstract

**Background:**

Non alcoholic fatty liver disease (NAFL) consists in the accumulation of fat vacuoles in the cytoplasm of hepatocytes. Many etiologic factors are associated with NAFL, such as, the metabolic syndrome factors, medications, bariatric surgery, nutritional disorders. However, very little information is available on the clinical relevance of this disorder as a health problem in the general population.

**Methods and design:**

The aim of the study is establish the risk factors most frequently associated with NAFL in a general adult population assigned to the primary care units and to investigate the relationship between each component of the metabolic syndrome and the risk of having a NAFL.

A population based case-control, observational and multicenter study will be carried out in 18 primary care units from the "Area de Gestión del Barcelonés Nord y Maresme" (Barcelona) attending a population of 360,000 inhabitants and will include 326 cases and 370 controls. Cases are defined as all subjects fulfilling the inclusion criteria and with evidence of fatty liver in an abdominal ultrasonography performed for any reason. One control will be randomly selected for each case from the population, matched for age, gender and primary care center. Controls with fatty liver or other liver diseases will be excluded.

All cases and controls will be asked about previous hepatic diseases, consumption of alcohol, smoking and drugs, and a physical examination, biochemical analyses including liver function tests, the different components of the metabolic syndrome and the HAIR score will also be performed. Paired controls will also undergo an abdominal ultrasonography.

**Discussion:**

This study will attempt to determine the factors most frequently associated with the presence of NAFL investigate the relationship between the metabolic syndrome and the risk of fatty liver and study the influence of the different primary care professionals in avoiding the evolution of the disease.

## Background

Non alcoholic fatty liver disease (NAFL) consists in the accumulation of fat vacuoles in the cytoplasm of hepatocytes and is characterized by the presentation of hepatic lesions similar to those made by alcohol in subjects without significant alcohol consumption. This term was introduced by Ludwig J in 1980 [1] and covers a wide spectrum of hepatic lesions including simple fatty liver, steatohepatitis with necroinflammatory changes and a variable degree of fibrosis which may finally progress to liver cirrhosis and, even hepatocarcinoma. However, there is little information on the clinical relevance of this disorder as a healthcare problem in the general population, since the studies published generally include a limited number of patients and the diagnosis is established on the basis of clear biochemical alterations and liver biopsy [2].

Many etiologic factors are associated with NAFL and are classified as primary, related to factors that cause an increase in insulin resistance (obesity, diabetes, dyslipemia), and the metabolic syndrome (obesity, type 2 diabetes, dyslipemia and arterial hypertension) and secondary to consumption of medications (amiodarone, tamoxifen, corticoids, estrogenos, nifedipine) to metabolic congenital or acquired alterations, nutrition, surgical procedures, and other toxics [3-7]. In clinical practice most patients with NAFL have obesity, type 2 diabetes or dyslipemia as the etiologic factor, with the association of several of these factors being frequent [8].

NAFL is possibly the most common cause of an elevation in transaminases in adults [9] and is considered the hepatic component of the metabolic syndrome [3]. The importance to detect patients with NAFL is to intervene in the associated factors and avoid evolution to more severe forms of the disease.

Fatty liver is an ever more prevalent disease involving 17 to 33% of the general population according to the different series and is increasing with the higher incidence of obesity [10]. A currently ongoing study in our reference area has found a prevalence of NAFL about 23% – 25% correlated with components of the metabolic syndrome, similar to other series [11]. In this study, the most important risk factor for NAFL is obesity, with 70% of overweight or obese patients presenting this disease.

Most patients with NAFL are asymptomatic. Diagnosis is made by presentation of increased transaminases, especially ALT, found during a health check up or during the study of some other manifestation of the metabolic syndrome. Diagnosis may also be suspected by accidental discovery of hepatomegaly or a radiological test carried out for another reason showing suggestive changes of fatty liver [8], although the final diagnosis should be confirmed by liver biopsy.

As we have seen the main risk factor for the development of NAFL is, therefore, obesity. It is well known that the increase in the number of obese persons within the general population has progressed up to the point that the healthcare authorities consider obesity to be one of the main healthcare problems for the near future and it has been made one of the priorities for intervention and investigation. In addition, patients with NAFL who are overweight/or obese are more to likely to develop steatohepatitis, with a percentage of these patients evolving to more severe forms of liver disease. We therefore believe that it is important to determine several indexes with the aim to establish which patients may evolve to more severe forms. These indexes are the HAIR score established by Dixon [12], the Mayo Clinic index (NAFLD fibrosis score) [13], among others. The HAIR score includes: insulin resistance defined as: type 2 diabetes with baseline glycaemia values greater than 110 mg/dl and less than 126 mg/dl and two of the following factors: arterial hypertension, triglycerides above 150 mg/dl and/or HDLc < 35 in males and < 39 in females, a waist/hip index > 0.90 in males and 0.85 in females and/or body mass index >30 kg/m2, and ALT levels > 40. Patients with a HAIR score ≥ 2 are considered to have a high probability of developing non alcoholic steatohepatitis.

The NAFLD fibrosis score combines: age, hyperglycaemia, body mass index, platelet count, albumin values and the AST/ALT ratio. The regression formula (risk score) for prediction of severity of fibrosis based on these 6 variables is: NAFL fibrosis score = -1.675 + 0.037 × age (years) + 0.094 × BMI (kg/m^2^) +1.13 × IFG/diabetes (yes = 1, no = 0) + 0.99 × AST/ALT ratio – 0.013 × platelet (×10^9^/l) – 0.66 × albumin (g/dl). Using the area under the ROC curve, 2 cutoff points were selected to identify the presence (greater than 0.676) and absence (lower than – 1.455) of significant fibrosis.

In addition to knowing the risk factors associated with the presence of NAFL, it is important to know the relationship between NAFL and the metabolic syndrome. The presence of the metabolic syndrome is increasing in the last years and is constituted by the presence of several factors in a same subject such as obesity, arterial hypertension, dyslipemia and glycaemia intolerance. Some criteria define the metabolic syndrome with the most commonly used being those of the World Health Organization (WHO) [14] modified by the European Group for the Study of Insulin Resistance (EGIR) [15], the criteria of the Adult Treatment Panel III (ATP-III) of the National Cholesterol Education Program (NCEP) [16], and those of the International Federation Diabetes [17]. These criteria include a combination of clinical and analytical parameters, mainly abdominal perimeter. Other parameters are the body mass index, insulin levels, arterial hypertension, glycaemia, HDL, triglycerides and microalbuminuria. So, the criteria of the WHO require the presentation of some alteration in carbohydrate metabolism, whether diabetes, abnormal glucose tolerance or resistance to insulin and two of the following criteria must also be taken into account: arterial hypertension > 140/90 mmHg, obesity (BMI = 30 kg/m^2^), hypertriglyceridemia ≥ 150 mg/dL or cHDL values < 35 in males and < 40 in females and microalbuminuria ≥ 20 μg/min. The EGIR has proposed several changes in the definition of the WHO, such as the presence of obesity (waist perimeter ≥ 94 cm in males and ≥ 80 cm in females), fasting plasma insulin determination and altered fasting glycaemia. The criteria of ATP-III of the NCEP are based on the presence of abdominal obesity (≥ 102 cm in males and ≥ 88 cm in females), hypertriclyceridemia (≥ 150 mg/dL), cHDL values < 40 mg/dl in males and < 50 mg/dl in females, arterial hypertension (≥ 130/85 mmHg) and altered fasting glycaemia (≥ 110 mg/dL). Finally, the criteria of the International Federation of Diabetes are based on the presence of abdominal obesity (≥ 94 cm in males and ≥ 80 cm in females), arterial hypertension (≥ 130/85 mmHg), altered fasting glycaemia (≥ 100 mg/dL), cHDL values (<40 mg/dl in males and <50 mg/dl in females), and hypertriclyceridemia (≥ 150 mg/dL). Given the importance of the metabolic syndrome we will analyse these three types of criteria in all the study subjects to determine which criteria identify the metabolic syndrome best.

## Objectives

### Main objectives

1. Establish the factors most frequently associated with the presence of NAFL in the people attending primary healthcare centers in the North Barcelona and Maresme area.

2. Evaluate the influence of each of the components making up the metabolic syndrome and the risk of having NAFL.

3. Identify the subjects who may progress to chronicity and the factors favouring progression.

### Secondary objective

1. Identify a cohort of patients with fatty liver with no evidence of previous liver disease to perform a prospective follow-up.

## Methods and design

We will carry out a observational analytical, population-based, multicentre study of cases and controls attending primary healthcare centers in the North Barcelona and Maresme area. The study has been approved by the Ethical Committee of Clinical Investigation, Jordi Gol i Gurina Foundation.

### Study subjects

This will be a multicentre study including the participation of 18 primary care teams covering a population of 360,000 inhabitants of an urban, and semi-rural zone of North Barcelona and Maresme, Spain.

#### Definition of cases

All adult patients fulfilling inclusion criteria in whom fatty liver is detected by means of ultrasonography performed for any reason in the reference Department of Radiology at the beginning of the study and have not received previous treatment for NAFL. Subjects will be considered as cases if they present fatty liver defined according to the standard criteria accepted by the American Gastroenterology Association: An Increase in hepatic echogenicity taking renal echogenicity as a reference, the presence of enhancement and lack of differentiation in periportal intensity and the vesicular wall due to great hyperechogenicity of the parenchyma. The degree of involvement will be standardized with a semiquantitative scale of the degree of hepatic enhancement.

#### Definition of controls

For every case an age (± 5 years) and sex-matched control will be selected from the same primary care centre by the same health care team. The controls will be selected from the SIAP (Primary Care Information System), a database that collects all the people that belong to one primary care center. This database in more complete and more updated than the population census. The controls will be invited to voluntarily participate and informed consent will be obtained for performing ultrasonography and analytical tests to ensure the absence of manifest hepatic disease. Subjects with normal ultrasonography results without alterations of hepatic enhancement will be considered as controls.

#### Inclusion criteria

Adults of both sexes from these Primary Care teams between the age 15 and 80 years who wish to voluntarily participate in the study and who have signed a written informed consent form to participate.

#### Exclusion criteria

Alcohol intake > 30 g/day in males and > 20 g/day in females. Presence of chronic liver disease. Presence of the hepatitis B virus surface antigen or the presence of virus hepatitis C antibodies. Subjects with conditions or diseases hindering data collection and follow up of the study such as incapacitating diseases, cognitive deterioration, institutionalized patients or subjects with no fixed address in any of the basic areas of the study. Subjects who do not provide written informed consent to participate in the study. The cases and controls with any exclusion criteria in their clinical history will not be invited to participate.

### Sample size

The prevalence of risk factors, except for alcohol, presented in the Spanish population in association with the presence of fatty liver described in the literature is the following: obesity 17.0%, diabetic mellitus 10.9%, and dyslipemia 18%. For a contrast of bilateral hypothesis and to detect an odds ratio of a minimum of 1.8 with exposure to one certain risk factor in the control group of at least 10%, with a alfa risk of 0.05 and a statistical power of 80%, 296 cases and 296 controls will be necessary. If we assume that 15% of the controls will have NAFL (not reflected in the clinical history) and that 10% of the cases and controls will have a high alcohol intake or the presence of hepatitis C virus antibodies or chronic liver disease (not reflected in the clinical history), 326 cases and 370 controls will be necessary.

### Variables

The following covariables will be evaluated in the patients fulfilling the inclusion criteria and who accept to participate in the study.

1. Sociodemographic variables: Age, sex, occupation, education and place of residence.

2. Clinical history including:

2.1. Personal history of liver disease, biliary lithiasis, surgical interventions.

2.2. Presence of comorbidities: Obesity and being overweight, type 2 diabetes, dyslipemia and arterial hypertension.

2.3. Alcohol intake: We will determine the type and quantity of alcoholic beverages consumed. The beverages consumed will be calculated per unit of standard beverage (glasses), differentiating the consumption between working days and the weekend. This will allow the calculation of the unit of standard beverage for each patient. Furthermore, the personal history of each case will be reviewed to rule out the possible history of high beverage consumption.

2.4. Smoking habit.

2.5. Habitual drug use: history of drug use during previous six months.

3. Physical examination:

3.1. Anthropometric data: weight, height, abdominal obesity and body mass index. The measurement of abdominal obesity will be done through the determination of the waist and hip circumferences using a tape measure (waist circumference will be considered the intermediate abdominal area perimeter between the last costal arch and the iliac crest measured in the horizontal plane, and hip circumference as that obtained in the plane of maximum relief of the gluteus muscles). The body mass index will be calculated according to the classical weight formula (kg/height m^2^).

3.2. Determination of arterial pressure: This will be determined with a validated automatic tensitometer with the subject resting. Three determinations will be carried out, separated by two minutes, and the final arterial pressure will be the average of the last two determinations discarding the first reading.

4. Analytical determinations:

4.1. Blood analyses will include: a complete haemogram, glycaemia, glycosylated haemoglobyn, urea, creatinine, uric acid, a lipid study (cholesterol, triglycerides, HDL, LDL), and liver function tests, including hepatitis markers (hepatitis B surface antigen virus and hepatitis C virus antibodies).

4.2. Baseline insulin levels will be determined by the immunochemoluminescence method.

4.3. Determination of insulin resistance will be established with the HOMA method (homeostasis model assessment). ([glycaemia (mmol/L) x insulin (mU/L)]/22.5).

4.4. A sample of the first urine in the morning will be taken to determine microalbuminuria.

4.5. Special analytical determinations: Frozen serum from all patients will be kept to determine different fibrotic markers (hyaluronic acid, leptin, adiponectin, TNF-alfa).

5. Determination of the different fibrotic indexes (HAIR score [12], NAFLD fibrosis score [13]).

6. Diagnosis of the metabolic syndrome: This will be made according to the criteria of the WHO modified by the European Group for the Study of Insulin Resistance (EGIR)[15], the Adult Treatment Panel III (ATP-III) of the National Cholesterol Education Program (NCEP) [16] and the International Federation of Diabetes [17].

### Control group

All the patients in this group will undergo ultrasonography for ruling out the presence of fatty liver.

### Plan of analysis

Data will be introduced into an ACCESS database and then analysed.

A descriptive univariate and bivariate statistical analysis will be performed, analysing the qualitative variables with the chi-square test and the quantitative variables with the Students-t test. Statistical significance will be established at 5% for all the bivariate analyses and contrasts will be considered at a bilateral level. Bivariate and multivariate analysis will be performed by means of a logistic regression model to observe the association of hypothetical risk factors of NAFL by calculating the odds ratio for each of these factors.

## Discussion

NAFL is a very prevalent disease and the present study will provide knowledge as to the most frequently associated factors in our reference area as well as the association between these factors and the presence of the metabolic syndrome, the prevalence of which is very high in Western countries. This will therefore provide appropriate data of our reference population regarding this increasingly more prevalent disease to sensitize healthcare professionals of the importance of the same and to insist about the risk factors, through norms of behavior, diet and drugs from our primary care centers to avoid the progression of NAFL to chronicity. Moreover, the study itself will generate a cohort of patients which, in successive years, will contribute knowledge regarding the natural history of NAFL.

On the other hand, health checkups are increasingly more common in the population as is the accessibility to both analytical and radiological diagnostic tests making it easier to detect these cases and consequently intervene. Moreover, in our country the diagnosis of NAFL is increasingly more frequent due to the interest given to this disease and, as has been mentioned, to the higher prevalence of obesity, manifesting during adolescence. Thus, although in the past NAFL was considered to be a disease mainly affecting the middle-aged, obese, and, in many cases, diabetic women, it has currently been shown to affect both sexes equally after adolescence [18].

The limitations of the studies of cases and controls are related to the representativeness of the cases selected for the study with respect to the whole population of cases. We can not make a random selection of the cases because, a priori, we do not know who this population is. With the design of this study we propose to select the cases in which a hepatic ultrasonography has been performed in the Radiology Department of reference in Primary Care of the population to study. This will ensure the origin of the populational basis of the cases and the accessibility to the main definitive diagnostic proof of the case. Cases which are not suspected, not diagnosed or who do not consult in the primary care may be under-represented but this possible selection bias will not be systematically introduced.

Another limitation of the study is related to the use of hepatic ultrasonography for the diagnosis of fatty liver. The gold standard for diagnosis of this disease is liver biopsy. Nonetheless, studies comparing the diagnostic utility of ultrasonography compared with liver biopsy have shown a sensitivity of greater than 90% and a specificity of greater than 80% for ultrasonography in detecting the presence of fatty liver. The main limitation seems to be the difficulty in detecting the presence of fatty liver when the infiltration is of less than 30% of the hepatic content. Another limitation of ultrasonography is the lack of information regarding the histologic changes associated with disease progression. To achieve this liver biopsy would be required which, in our case would be referred to the reference hospital on both clinical and biochemical suspicion of progression. Therefore, ultrasonography of the liver is currently the test of reference for the detection of fatty liver at a population level [19].

Non alcoholic fatty liver disease is a health problem which, in association with other frequent conditions, is very prevalent in our centres. Although until recently the presence of this disease was approached as a minor problem, it must be taken into account that it may be potentially serious because of its possible evolution to chronicity and to hepatic cirrhosis. On the other hand, its detection today in primary care centers is relatively easy and accessible, and more importantly, a multifactorial intervention on the risk factors may favour its remission and thereby avoid the appearance of more severe hepatic complications.

## Competing interests

The authors declare that they have no competing interests.

## Authors' contributions

LCR, JALL, PTM, MBP, EAF, M^a^AALL, LMO and GPB participated in the design of the study; LCR, MBP, EAF, M^a^AALL, ATC, MAP, SCC and JBS contributed to the coordination study; DMB, JDCC, CSG performed the ecographies; AAT performed biochemical tests; GPB participated in the statistical calculations. All the authors have read, revised and approved the final manuscript.

## Pre-publication history

The pre-publication history for this paper can be accessed here:


